# Networks of cortical activity show graded responses to perinatal asphyxia

**DOI:** 10.1038/s41390-023-02978-4

**Published:** 2023-12-22

**Authors:** Timo Syvälahti, Anna Tuiskula, Päivi Nevalainen, Marjo Metsäranta, Leena Haataja, Sampsa Vanhatalo, Anton Tokariev

**Affiliations:** 1 Department of Clinical Neurophysiology, Children´s Hospital, and Epilepsia Helsinki, full member of ERN EpiCare, HUS Medical Imaging Center, University of Helsinki and Helsinki University Hospital (HUH), Helsinki, Finland; 2 BABA center, Pediatric Research Center, Children’s Hospital, University of Helsinki and HUH, Helsinki, Finland; 3 Department of Pediatrics, Children’s Hospital, University of Helsinki and Helsinki University Hospital (HUH), Helsinki, Finland; 4 Department of Pediatric Neurology, Children’s Hospital, University of Helsinki and Helsinki University Hospital (HUH), Helsinki, Finland

## Abstract

**Background:**

Perinatal asphyxia often leads to hypoxic-ischemic encephalopathy (HIE) with a high risk of neurodevelopmental consequences. While moderate and severe HIE link to high morbidity, less is known about brain effects of perinatal asphyxia with no or only mild HIE. Here, we test the hypothesis that cortical activity networks in the newborn infants show a dose-response to asphyxia.

**Methods:**

We performed EEG recordings for infants with perinatal asphyxia/HIE of varying severity (*n* = 52) and controls (*n* = 53) and examined well-established computational metrics of cortical network activity.

**Results:**

We found graded alterations in cortical activity networks according to severity of asphyxia/HIE. Furthermore, our findings correlated with early clinical recovery measured by the time to attain full oral feeding.

**Conclusion:**

We show that both local and large-scale correlated cortical activity are affected by increasing severity of HIE after perinatal asphyxia, suggesting that HIE and perinatal asphyxia are better represented as a continuum rather than the currently used discreet categories. These findings imply that automated computational measures of cortical function may be useful in characterizing the dose effects of adversity in the neonatal brain; such metrics hold promise for benchmarking clinical trials via patient stratification or as early outcome measures.

**Impact:**

- Perinatal asphyxia causes every fourth neonatal death worldwide and provides a diagnostic and prognostic challenge for the clinician.
- We report that infants with perinatal asphyxia show specific graded responses in cortical networks according to severity of asphyxia and ensuing hypoxic-ischaemic encephalopathy.
- Early EEG recording and automated computational measures of brain function have potential to help in clinical evaluation of infants with perinatal asphyxia.

## Introduction

Perinatal asphyxia affects 4 million newborn infants each year causing every fourth neonatal death and around 1 million infants with lifelong neurological impairments.^1,2^ Perinatal asphyxia commonly leads to hypoxic-ischaemic encephalopathy (HIE) ranging from mild to severe,^3^ occurring in 1,5 per 1000 live births in developed countries,^4^ and carrying a 37% overall risk of neurological sequelae.^5^ It is well established that infants surviving moderate or severe HIE may develop severe impairments such as cerebral palsy or significant cognitive impairment. However, less is known about mild HIE and even less about perinatal asphyxia without clinical HIE (hereafter referred to as PA), though recent evidence suggests that they may also associate with later neurodevelopmental problems.^6–9^ Indeed, recent work on structured neurological examinations, magnetic resonance imaging (MRI) and neurological outcomes have supported the clinically intuitive idea that different grades of aspyxia/HIE are better described as a continuum rather than distinct groups.^10^

While the effects to EEG background are well characterized,^11–15^ it is poorly known how neuronal activity networks are affected in infants with different grades of HIE. Neuroimaging studies have indicated incremental structural changes with increasing HIE severity,^10^ and recent works using EEG have reported significant network effects in infants with moderate-to-severe HIE.^16–18^ However, it remains unknown whether cortical network activity is affected in infants with milder forms of asphyxia/HIE, which account for the majority of infants that are medically cared for in the NICUs after perinatal asphyxia.

In addition to the routine clinical reviews,^19^ EEG can be used for examining wide-scale brain networks in multiple ways that reflect different underlying mechanisms of cortico-cortical interactions. The best characterized analysis frameworks include the three intrinsic coupling modes in neuronal interactions: (i) phase–phase correlations (PPCs)^20,21^ that reflect neuronal interactions with high temporal resolution, (ii) amplitude–amplitude correlations (AACs)^22,23^ representing less specific co-activations between cortical regions, and (iii) phase–amplitude correlations (PACs)^24^ that relate to cross-frequency interactions within or between cortical areas. In addition, activation synchrony index (ASI) can be used as a computational estimate of the clinically well-established interhemispheric synchrony.^25^ All of these measures correlate with physiological factors, such as vigilance state or maturation,^26–28^ but are also sensitive to medical adversities and treatments, such as prematurity,^28–31^ HIE,^16^ fetal drug exposure,^32–34^ and early nursing interventions.^26^

Here, we set out to study cortical activity networks in healthy control infants and infants suffering from perinatal asphyxia with or without later development of mild or moderate HIE. We hypothesized that different grades of perinatal asphyxia, with or without HIE, form a continuum that exhibits a dose-response in the changes of cortical activity networks when estimated with a battery of well-established EEG network measures.

## Materials and methods

**Fig. 1:**
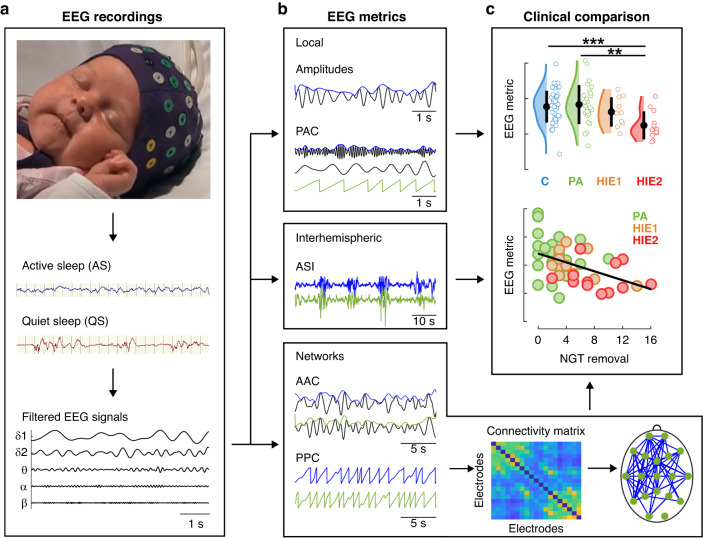
Overview of the study. **a** Sleep EEG data was collected from both asphyxic and control infants. EEG data was visually scored into active sleep (AS) and quiet sleep (QS). For analysis, we used signals from 19 electrodes filtered to five frequencies. **b** Five distinct EEG metrics were analyzed for all infants and study groups: amplitudes, phase-amplitude correlations (PACs), activation synchrony index (ASI), amplitude-amplitude correlations (AACs), and phase–phase correlations (PPCs). **c** All EEG metrics were compared between control infants and infants with different severity of asphyxia/HIE and correlated with early clinical recovery, namely, removal of nasogastric tube (NGT), e.g., the time that infants started normal feeding. AAC and PPC metrics both yeilded 19 ×19 × 5 × 2 (electrodes × electrodes × frequencies × sleep states) connectivity matrices, which were visualized with scalp topographical plots depicting connections with significant difference between groups. C controls, PA perinatal asphyxia without HIE, HIE1 mild hypoxic ischemic encephalopathy, HIE2 moderate hypoxic ischemic encephalopathy.

Overview of the methods and analytical flow in the study is shown in Fig. 1 and described in detail in the following sections.

### Patients

For the present study, we collated EEG data from a clinical cohort of perinatal asphyxia and a cohort of healthy control infants. Clinical characteristics of the groups are presented in Table 1. The infants in the perinatal asphyxia cohort (*N* = 52; gestational age ≥37 weeks) were prospectively recruited from the neonatal wards of the Helsinki University Hospital (Helsinki, Finland) and Jorvi Hospital (Espoo, Finland) between September 2016 and September 2020. The infants were identified as presenting with clinical signs of perinatal asphyxia without other apparent reason for distress at birth. The criteria for asphyxia were umbilical arterial cord pH ≤ 7.10, 1-min Apgar score ≤ 6, need for assisted ventilation, and/or cardiopulmonary resuscitation at birth. The exclusion criteria were lack of neonatal EEG-recording, congenital anomaly, chromosomal abnormality, another neurological condition, or infection. As we focused on milder sequalae of perinatal asphyxia, we also excluded infants with severe HIE. The attending neonatologist decided on treatment with therapeutic hypothermia based on clinical guidelines^35^ during the first six hours of life. Further description of the inclusion and exclusion criteria has been reported in our previous publication of this cohort.^35^

**Table 1:** Comparison of clinical characteristics in the cohorts.

		Control group	Study group (*n* = 51)			*p* value*
		C (*n* = 53)	PA (*n* = 29)	HIE1 (*n* = 10)	HIE2 (*n* = 12)	
Male sex (*n*, %)		30 (57 %)	14 (48 %)	5 (50 %)	9 (75 %)	0.477
GA, weeks (median, IQR)		40 + 0 (39 + 4 – 40 + 5)	40 + 5 (39 + 6 – 41 + 5)	40 + 3 (38 + 1 – 41 + 6)	40 + 0 (38 + 5 – 41 + 2)	**0.035**^**a**^
Birth weight, grams (median, IQR)		3520 (3250–3710)	3570 (3300–3970)	3310 (2750–3700)	3490 (2990–3760)	0.228
Apgar (median, IQR)	1 min	9 (9–9)	3 (2–5)	2 (2–5)	3 (1–3)	**<0.001**^**abc**^
	5 min	10 (10–10)	6 (4–7)	5 (3–6)	4 (3–5)	**<0.001**^**abc**^
	10 min	10 (10–10)	7 (7–8)	6 (6–7)	6 (4–7)	**<0.001**^**abcd**^
Cord arterial pH (median, IQR)		7.27 (7.23–7.32)	7.06 (6.98–7.10)	7.00 (6.97–7.10)	7.10 (7.04–7.17)^f^	**<0.001**^**abc**^
Cord arterial BE (median, IQR)		−3.0 (−5.8 to (−1.4))	−8.7 (−10.2 to (−6.3))	−11.8 (−12.6 to (−8.1))	−8.1 (−11.3 to (−4.9))^f^	**<0.001**^**abc**^
Abnormal neurological status, age <3 h (*n*, %)		0 (0 %)	21 (72 %)	10 (100 %)	12 (100%)	**<0.001**^**abc**^
Clinical seizures throughout hospital stay (clinical symptoms or need of antiepileptic medication due to repetitive seizures in the aEEG) (*n*, %)		0 (0 %)	0 (0 %)	1 (10 %)	5 (42%)	**<0.001**^**cd**^
Therapeutic hypothermia (*n*, %)		0 (0 %)	0 (0 %)	0 (0 %)	9 (75%)	**<0.001**^**cde**^
Need of NG tube feeding (*n*, %)		0 (0 %)	21 (72 %)	10 (100 %)	12 (100%)	**<0.001**^**abc**^
Days of NG tube feeding (median, IQR)		0 (0–0)	2 (0–4)	4 (3–7)	8 (5–12)	**<0.001**^**abcd**^

*P values for the categorical variables are calculated with Fisher’s exact and for the continuous variables are calculated with Kruskal–Wallis and Wilcoxon Rank Sum tests with Bonferroni correction. Values <0.05 are bolded.

^a^C vs. PA.

^b^C vs. HIE1.

^c^C vs. HIE2.

^d^PA vs. HIE2.

^e^HIE1 vs. HIE2.

^f^Cord arterial sample was obtained only in 8/12 cases, which may influence the results.

The infants of the asphyxia cohort were divided into three groups: perinatal asphyxia without HIE (PA), mild HIE (HIE1) or moderate HIE (HIE2), as defined by the modified Sarnat score3 together with the amplitude-integrated electroencephalography (aEEG) findings during the first 24 h of life. To ensure consistent diagnostic categorization, all clinical data was retrospectively re-evaluated by a team including a neonatologist (MM), a pediatric neurologist (LH) and a pediatrician in training (AT).

The control group (C; *N* = 53) included full-term infants (gestational age ≥37 weeks) born from an uneventful pregnancy. These infants were identified from our research database (see also^26,28–30^) using the following inclusion criteria: sufficient amount of good quality EEG for the analyses, no medical events between birth and discharge from the hospital, as well as normal umbilical cord blood gases and a 1-min Apgar score ≥8.

### Measures of early clinical recovery

For a bedside surrogate of early clinical recovery, we considered the number of days needed for feeding with nasogastric tube (NGT) before commencing full oral feeding as taken from the medical records. Time to NGT weaning was chosen because it is readily available worldwide, and it was according to our yet unpublished work found to provide a transparent and clinically meaningful bedside proxy measure of recovery. NGT removal time correlates with EEG abnormalities,^36^ and also the severity of neurological symptoms.

### EEG recordings

EEG was recorded using an EEG cap (sintered Ag/AgCl electrodes; Waveguard, ANT-Neuro, Germany), and the EEG analysis was done from the 19 signals recorded at the standard 10-20 locations (see also^20,21^). The signals were collected using either an Eego EEG amplifier (ANT-Neuro, Germany) at a sampling rate of Fs = 500 Hz, or a NicoletOne EEG system (Cardinal Healthcare/Natus) at a sampling rate of Fs = 2000 Hz. Additional polygraphy channels were added when possible to aid in later vigilance state (active or quiet sleep stages) detection during visual EEG review as previously described in detail.^29,30,32^ Electrode impedances were below 10 kOhm before recordings, and signal quality was constantly monitored. For further details of neonatal multichannel EEG recording see http://www.helsinki.fi/science/eeg/videos/nemo/. Recordings were done at a postmentrual age of 42.0 ± 1.0 weeks for controls, 43.2 ± 1.5 weeks for PA group, 42.2 ± 2.0 weeks for HIE1 group, and 40.6 ± 1.5 weeks for HIE2 group (mean ± standard deviation).

### EEG analyses

#### Overview of the EEG analyses

We analyzed five EEG measures for all infants and sleep stages: (i) Frequency-specific amplitudes were estimated as a measure of the overall oscillatory activity within each frequency band of interest. (ii) The ASI was computed to estimate the level of interhemispheric synchrony to provide a clinically interpretable comparison to prior EEG studies with visual EEG review.^25^ The (iii) PPC and (iv) AAC measures were computed to obtain pairwise interactions between cortical regions via these intrinsic modes of connectivity.^20–23^ (v) PAC was computed to assess local cross-frequency interactions that are considered to reflect interactions within intracortical networks.^24^ These measures were compared between infant groups allowing assessment of a possible dose-response of asphyxia/HIE severity in the brain networks. To further test our hypothesis regarding the dose response type of effect of asphyxia on cortical activity, all EEG measures were correlated with early clinical recovery assessed by the NGT removal time. A more detailed description of these measures is presented below.

#### Visual inspection

All EEG data were converted to European Data Format (EDF) for visual review and epoch selection in a clinical software (NicOne Reader, Natus). One trained expert (TS) visually inspected the EEG data, scored sleep states based on standard criteria, and selected a total of 3 min of artifact-free data of both active (AS) and quiet sleep (QS). In QS EEG consisted of high voltage low frequency activity or tracé alternant and respiration pattern was regular. In AS EEG consisted of low voltage high frequency activity and respiration pattern was irregular. Few infants had EEG that met the selection criteria only during one sleep state (*N* = 5 subjects had only AS data and *N* = 4 had only QS data), and they were included into the analysis of the corresponding state. One infant with PA was excluded from the analysis due to poor EEG quality.

#### EEG preprocessing

We performed all subsequent EEG analyses using custom-made Matlab scripts (MATLAB R2021b, MathWorks, Natick, MA), and using the same 19 EEG channels that were common to the entire study cohort (Fp1, Fp2, F7, F3, Fz, F4, F8, T7, C3, Cz, C4, T8, P7, P3, Pz, P4, P8, O1, and O2). The EEG signals were first band-pass filtered within 0.4–45 Hz frequency range and down-sampled to Fs = 100 Hz to reduce computational load. Except for the ASI computation, we then filtered the signals into 5 frequency bands of interest: low delta (*δ*1) 0.4–1.5 Hz, high delta (*δ*2) 1.5–4 Hz, theta (θ) 4–8 Hz, alpha (α) 8–13 Hz, beta (β) 13–22 Hz. All band-pass filtering was implemented by applying in series a combination of low-pass and high-pass Butterworth filters with the corresponding cut-off frequencies. Filtering was done offline in forward-inverse direction to avoid phase distortions. The filter banks were designed as steep as stability allowed (stop-band frequencies were in a range of 0.1–0.4 Hz from the cut-offs) and attenuation after filtering was at least 30 dB.

Finally, we re-referenced EEG signals to Laplacian montage (also known as *current source density, CSD*) using spline spherical interpolation method published online as a MatLab toolbox (http://psychophysiology.cpmc.columbia.edu/Software/CSDtoolbox). We used CSD with the following parameters: smoothing constant λ = 0 and head radius = 5 cm.^37–39^

#### Frequency-specific amplitudes

The mean of amplitude envelopes was computed from band-filtered EEG signals using Hilbert transform.^40^ Global amplitudes were taken as the average over channels, separately for each frequency band.

#### Activation synchrony index (ASI)

Interhemispheric synchrony was evaluated using an established quantitative measure, Activation Synchrony Index (ASI),^25^ which quantifies the co-occurrence of bursting activity between two EEG signals. It is particularly interpretable for assessing regional interactions during intermittent EEG activity, such as what is seen during QS in the infants. Here, we computed ASI for seven symmetric across hemispheres pairs of electrodes, five single electrode pairs using Laplacian montage (Fp1 vs Fp2, F3 vs F4, C3 vs C4, P3 vs P4, O1 vs O2), and two resembling the clinical visual aEEG review using bipolar montage (F3-P3 vs F4-P4, C3-P3 vs C4-P4). All analyses included 3-min epochs of only QS.

#### Large-scale PPC and AAC networks

The large-scale cortical network interactions were examined using phase–phase correlations (PPCs) and amplitude-amplitude correlations (AACs) between pairs of EEG signals. PPCs are thought to depict sub-second modulations in neuronal activity, whereas AACs are considered to reflect co-modulations of overall cortical activity in time frames of seconds.^20–22^ PPCs and AACs were computed for each pair of EEG signals (total N = 171 pairs), for each frequency and each sleep state, which resulted in *N* = 10 connectivity matrices in each infant per modality. PPCs were estimated using the debiased version of weighted phase lag index, wPLI:^41^

$${{wPLI}}=\frac{\left|E\left[{{{I}}m}\left({S}_{{xy}}\right)\right]\right|}{E\left[\left|{{{Im}}}\left({S}_{{xy}}\right)\right|\right]}$$

where, E[] stands for expectation, Im() shows imaginary part, and S_xy_ is a cross-spectral density of two time series x and y. In turn, AACs were computed as Pearson correlation coefficient between the amplitude envelopes of two mutually orthogonalized signals, oCC:^42^

$${{oCC}}={{corr}}(x,{y}^{* })$$

where, y* is the version of the original signal y which was orthogonalized relative to signal *x* as following: y* = *y* − *β·x*, and *β* is the regression coefficient. Orthogonalization was done in both directions: first, *y* relative to x, and then x relative to y. The average of two resultant oCC values was taken as a measure of AAC. We chose these connectivity estimators to minimize potential effects caused by volume conduction.^43,44^ The networks were represented as connectivity matrices in both PPC and AAC estimates, which allowed comparison of network strengths at different spatial levels (global, regional, and connection-wise).

#### Local cross-frequency interactions

The local cortical cross-frequency interactions were estimated with phase-amplitude correlations (PACs), which are thought to reflect local connectivity between cortical layers.^24^ PACs have been shown to correlate with maturation and vigilance states in infants.^27,45^ Here, we estimated PAC as previously described:^27^ we computed the phase locking value between the phase of low-frequency (nesting) oscillations (${{{{{{\rm{\varphi }}}}}}}_{L}$) and the phase of the amplitude envelope of the higher frequency (nested) oscillation (${{{{{{\rm{\varphi }}}}}}}_{H}$) after filtering with the same filter used for the nesting frequency:

$${{PAC}}=\frac{1}{N}\left|\mathop{\sum }\limits_{k=1}^{N}{e}^{\,j\left({{{{{{\rm{\varphi }}}}}}}_{L[k]-}{{{{{{\rm{\varphi }}}}}}}_{H[k]}\right)}\right|$$

We used low delta as nesting frequency and theta, alpha, and beta as nested frequencies. The PACs were first computed separately for each channel and then averaged to obtain a global measure of PAC.

### Statistical analysis

We performed the statistical analyses with MATLAB R2021b. As the data were mostly not normally distributed, we used nonparametric statistics in all comparisons. The age difference (*p* < 0.001, Kruskal–Wallis test) between groups at EEG may affect the group comparisons,^26,27^ hence we corrected different postmenstrual ages at the time of the EEG recording as follows. First, we used linear fitting to compare age against each EEG metric. Then, we computed Studentized residuals as a measure of age-corrected values for that metric, multiplied Studentized residuals with standard deviation and added mean to get results in the original scale.^26^ Before age-correction we found many EEG measures to correlate significantly with postmenstrual age;^27^ these correlations were eliminated after the correction procedure. All EEG metrics (amplitudes, ASI, AAC, PPC, PAC) were first compared between all four infant groups (C, PA, HIE1, HIE2) with Kruskal–Wallis test. In case of significant findings, pairwise *post-hoc* comparisons were done with Wilcoxon Rank Sum test. In AAC and PPC post-hoc analysis, effect size was estimated using rank-biserial correlation ranging from −1 to 1, averaged across all edges. For evaluating independence of our metrics, we analyzed correlations between all EEG metrics (see Supplementary Fig. 1). For the asphyxia/HIE group, we computed Spearman correlation coefficients between NGT removal day and each of the EEG metrics. For correcting multiple comparisons, we used False Discovery Rate correction with Benjamini-Hochberg procedure.^46^ We set False Discovery Rate at 0.05.

## Results

### Frequency-specific amplitudes

**Fig. 2:**
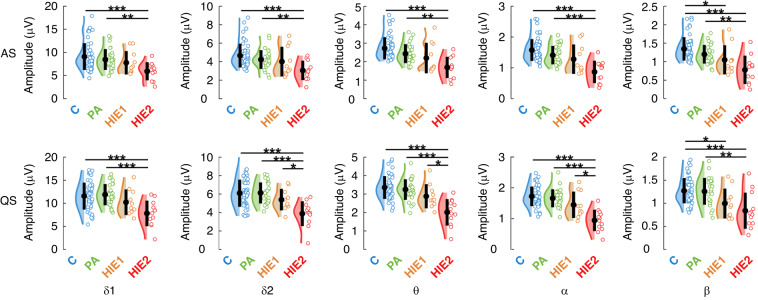
Effects of asphyxia on global frequency-specific amplitudes in different infant groups. Significant group differences were seen at all frequency bands and in both sleep states, and the pairwise post-hoc test indicated significant differences in almost half of the tested group pairs. Asterisks stand for corrected *p* values: *p* < 0.001, *p* < 0.01, and *p* < 0.05 in the pairwise comparisons. *δ*1: low delta, *δ*2: high delta, θ: theta, α: alpha, β: beta. C controls, PA perinatal asphyxia without HIE, HIE1 mild hypoxic ischemic encephalopathy, HIE2 moderate hypoxic ischemic encephalopathy.

Global amplitudes of cortical EEG activity were significantly different between infant groups in both sleep states (Fig. 2; Supplementary Fig. 2). There was a clear, systematic trend with higher HIE grade linking to lower amplitudes at all oscillatory frequencies. Group wise comparison showed that C and PA groups had significantly higher EEG amplitudes compared to the HIE2 group in both sleep states and in all frequencies (*p* < 0.01), where as C infants showed higher amplitudes compared to the HIE1 group at beta frequency during both sleep states (*p* < 0.01).

### Activation synchrony index (ASI)

**Fig. 3:**
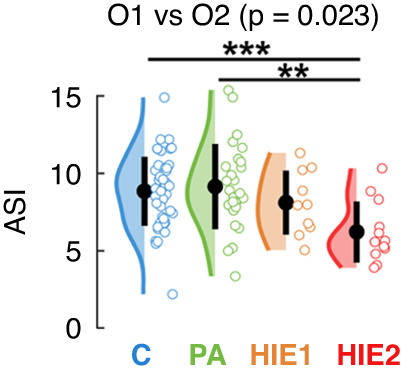
Effects of asphyxia on interhemispheric synchrony. We found a significant group difference in the occipital interhemispheric comparison (O1 vs O2; Laplacian reference). Higher ASI values indicate higher synchrony between hemispheres. Asterisks stand for *p* < 0.01 and *p* < 0.05 in pairwise comparisons. *P*-values are Benjamini-Hochberg-corrected. C controls, PA perinatal asphyxia without HIE, HIE1 mild hypoxic ischemic encephalopathy, HIE2 moderate hypoxic ischemic encephalopathy.

The measure of interhemispheric synchrony, ASI, showed an expected^25^ general decline towards higher HIE grades; however, the group difference was statistically significant only between the occipital channels (O1 vs O2; p = 0.023, Fig. 3), and only between HIE2 and the C/PA groups (see also Supplementary Fig. 3).

### Large-scale PPC and AAC networks

**Fig. 4:**
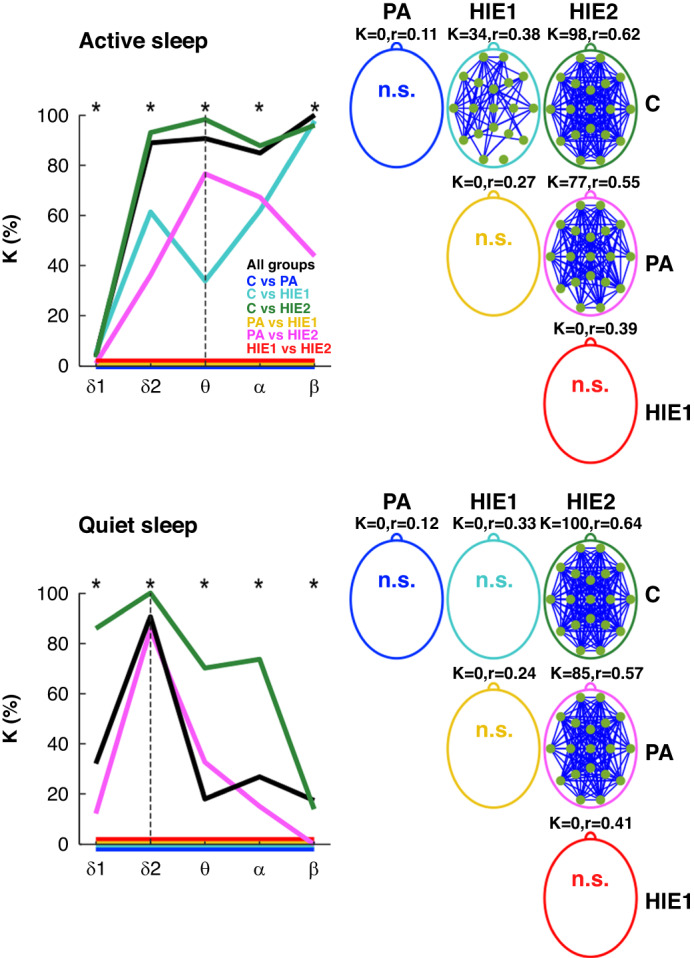
AAC results during active and quiet sleep periods. On the left, comparison of amplitude-amplitude correlations (AACs) between infant groups are shown for the proportion of edges (connections; K%) out of all connections within each frequency band. Asterisks stand for *p*-values < 0.05 in comparisons of all groups. On the right, the topographical plots illustrate the distribution of edges showing significant differences between all group pairs. For active sleep the comparisons are shown for theta frequency and for quiet sleep for high delta frequency. Blue color indicates that the group on top had higher AACs. Hence, in the examples the clinically worse group always showed higher AAC levels. *K*-values (*K*) and effect sizes (*r*) are shown above each topographical plot. “N.s.” indicates no edge-wise significance between groups. *δ*1: low delta, *δ*2: high delta, θ: theta, α: alpha, β: beta. C controls, PA perinatal asphyxia without HIE, HIE1 mild hypoxic ischemic encephalopathy, HIE2 moderate hypoxic ischemic encephalopathy.

There were significant group differences in the AAC networks at all frequencies (Fig. 4), however the proportion (*K*) of such cortical connections varied largely between frequencies and sleep states (Fig. 4). During AS, the AAC network strengths were widely (*K* = 50–100%) inversely linked to HIE severity at frequencies > 1.5 Hz, and with a couple of connections (*K* = 4%) in low delta frequency (Fig. 4a). In all frequencies, HIE1 and HIE2 infants showed higher levels of AAC compared to C, and HIE2 infants also to PA. During QS, the HIE2 infants showed higher levels of AAC connectivity at frequencies <13 Hz compared to PA, and in all frequencies compared to C (Fig. 4b).

There were no group differences found in the PPC networks at any frequency band.

### Local cross-frequency interactions

**Fig. 5:**
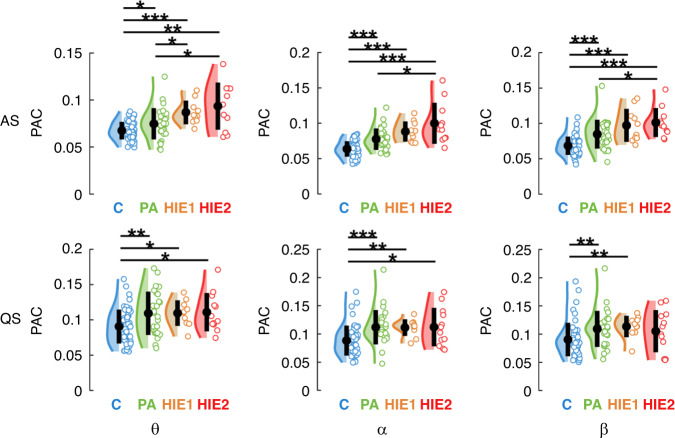
Effects of asphyxia on phase-amplitude correlations (PACs). PACs were compared between groups for different nested frequencies (θ: theta, α: alpha, β: beta) in active (AS) and quiet sleep (QS). Higher values indicate stronger PACs. Asterisks stand for *p* < 0.001, *p* < 0.01, and *p* < 0.05 in pairwise comparisons. *P*-values are Benjamini–Hochberg corrected. C controls, PA perinatal asphyxia without HIE, HIE1 mild hypoxic ischemic encephalopathy, HIE2 moderate hypoxic ischemic encephalopathy.

Globally pooled levels of PAC showed statistically significant differences between the groups in both sleep states and at all frequencies, with a consistent increase with increasing severity of HIE (Fig. 5; Supplementary Fig. 4).

### Correlations of cortical activity to early clinical recovery

**Fig. 6:**
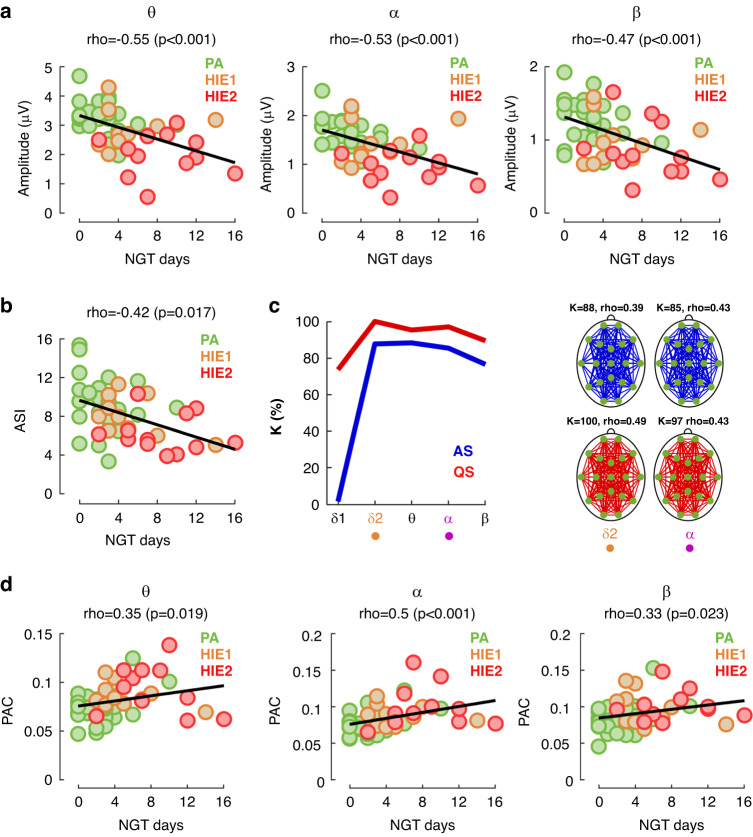
Correlations between cortical activity measures to early clinical recovery measured by the NGT removal time. **a** Significant correlation was observed between NGT removal time and the EEG amplitudes in quiet sleep, as well as **b** between NGT removal time and ASI (O1 vs O2 derivation). **c** AAC levels were significantly correlated to NGT removal time in nearly all (*K* = 80–100%) connections and the spatial topographies show uniform distributions of the significant connections. **d** PAC levels in active sleep and NGT removal time showed significant correlations. See also Supplementary Fig. 5 for results in all sleep states and ASI derivations. The colored dots represent individual infants: green = perinatal asphyxia without HIE (PA), yellow = mild HIE (HIE1), red = moderate HIE (HIE2). *ρ* = Spearman correlation coefficient. *δ*1: low delta, *δ*2: high delta, θ: theta, α: alpha, β: beta.

All EEG metrics other than PPC correlated significantly with early clinical recovery measured with NGT removal time. Longer need of NGT (i.e. poorer recovery) was linked to lower EEG amplitudes and ASI, while shorter need of NGT (i.e. better recovery) was linked to lower AAC and PAC levels (Fig. 6; Supplementary Fig. 5). Taken together HIE group-dependent effects in EEG measures are fully compatible with the correlations between EEG measures and the NGT removal times.

## Discussion

Our data show that measures of cortical activity, both locally and in the large-scale cortical networks, exhibit robust correlations to the clinical grades of perinatal asphyxia/HIE. Most such effects correlate with the asphyxia/HIE severity, and moreover, they also correlate strongly with an early bedside measure of clinical recovery, time to attain full oral feeding. The findings are largely compatible with prior studies reporting EEG findings from either visual review or selected computational EEG metrics.^13,16,47–49^ However, we extend prior literature by systematically characterizing the effects of asphyxia on different neuronal coupling modes, i.e. cortical network activity, and their relationship to early clinical recovery.

Previous studies have shown various effects of early adversity on cortical activity near term age: for instance, *in utero* exposure to anti-depressant drugs associates with lower levels of EEG amplitudes, PAC, and ASI as compared to healthy infants.^33^ Exposure to antiepileptic drugs, in turn, was related to fronto-occipital gradient in ASI levels.^32^ Cortical large-scale PPC and AAC networks, and PACs, also change between vigilance states and during maturation around the first weeks of life,^27^ while the strength of PPC networks in ex-preterm infants is linked to both early and longterm neurodevelopmental performance; all these suggest that measures of cortical activity networks may have utility as an early functional biomarker.^30^

To our knowledge, only a couple of previous publications have investigated the changes in cortical activity networks due to mild or moderate HIE.^16–18,50^ Although previous studies differ from ours with respect to the exact EEG metrics, the findings are generally in line with our work showing large effects by moderate HIE, in particular in measures of amplitudes, ASI, AACs, and PAC.

Our findings show dose-response^51^ in EEG with respect to increasing severity of asphyxia/HIE, in particular in the global levels of amplitudes and PAC (Figs. 2 and 5). It is conceivable that the effects of perinatal asphyxia and/or ensuing encephalopathy are better characterized as a spectrum rather than the discrete categories commonly adopted in the clinical work for practical convenience. Such spectrum of severity is also supported by the recently published cohort studies with neurobehavioral and MRI characterizations.^8,9^ The EEG measures reported in the present study could potentially be used as a complementary metric that may facilitate construction of an improved continuous measure of asphyxia/HIE severity.

The PA infants also showed small, yet statistically significant effects in their PAC levels compared to controls (Fig. 5). PAC is considered to reflect neuronal interactions across cortical layers,^24^ hence the interpretation of the finding is that even perinatal asphyxia without clinical signs of HIE may re-organize the intracortical neuronal circuits, an effect that may well be significant for the ongoing, activity-dependent neurodevelopment.^52,53^ An effect of PA on early neurodevelopment is also supported by the deviant neurological outcome at few months of age;^10^ the outcome results have varied between studies,^8,54^ and our results are only coming in the near future.

Here we used time to attain full oral feeding as an early outcome measure because infants with HIE often present with some degree of feeding impairment.^36,55,56^ Oral feeding is a complex sensorimotor process that requires coordination and precise timing of sucking, swallowing, and breathing. This process is regulated by a neural network requiring simultaneous function of numerous distinctive brain areas,^57^ and therefore time of NGT removal reflects readiness of the infant to full oral feeding. Importantly, our yet unpublished work shows that NGT removal time correlates significantly with other neurodevelopmental measures, such as the score of the Hammersmith Infant Neurological Examination at three months, or deep gray and white matter scores in the neonatal MRI. Our present results show that NGT removal time correlates significantly with a range of EEG-based measures of cortical network activity (amplitudes, AAC, PAC in active sleep and ASI). Our findings support prior reports^57^ that neonatal feeding impairments measured in such a straightforward manner at bedside can be used as indicators of neonatal brain dysfunction, and in this context, to see a dose-response effect of perinatal asphyxia.

Our work is limited by the cohort size, especially when comparing the subgroups of perinatal asphyxia/HIE; the present results will need validation from larger, prospective cohorts, where statistical power can be improved by only studying metrics that were found significant in the present study. Additionally, postmenstrual age at the time of EEG recording might present a possible confounding factor. Our prior studies have generally involved infants at 41-43 weeks postmenstrual age, showing some changes even within this time window.^27^ Due to the subtle age differences between the infant groups, we applied statistical corrections for age.

A key strength of this study is the use of systematic evaluation of EEG measures that cover a wide spectrum of cellular and network mechanism of cortical neuronal activity. Prospective design of the clinical study cohort enabled recruitment of the yet rarely studied patient group, infants with mild HIE and even infants with asphyxia without HIE. These infants are usually not studied with EEG or standardized neurological examinations in routine clinical setting. Collating the wide spectrum of clinical presentations from PA to HIE2 allowed assessing the dose-response type effect of asphyxia on the neonatal cortical function.

### Supplementary Information

Supplementary Material

## Data Availability

Original data can not be shared because it contains sensitive patient data. However, adjacency matrices and other derivatives can be made available by a reasonable request from the authors.
