# Supplementary material for: Networks of cortical activity show graded responses to perinatal asphyxia
Source: Pediatr Res. 2023 Dec 22;96(1):132–40. doi: 10.1038/s41390-023-02978-4 (PMC11258028; doi:10.1038/s41390-023-02978-4)
Supplement: Supplementary file 1 — Supplementary Material [file 41390_2023_2978_MOESM1_ESM.pdf]

SUPPLEMENTARY MATERIAL

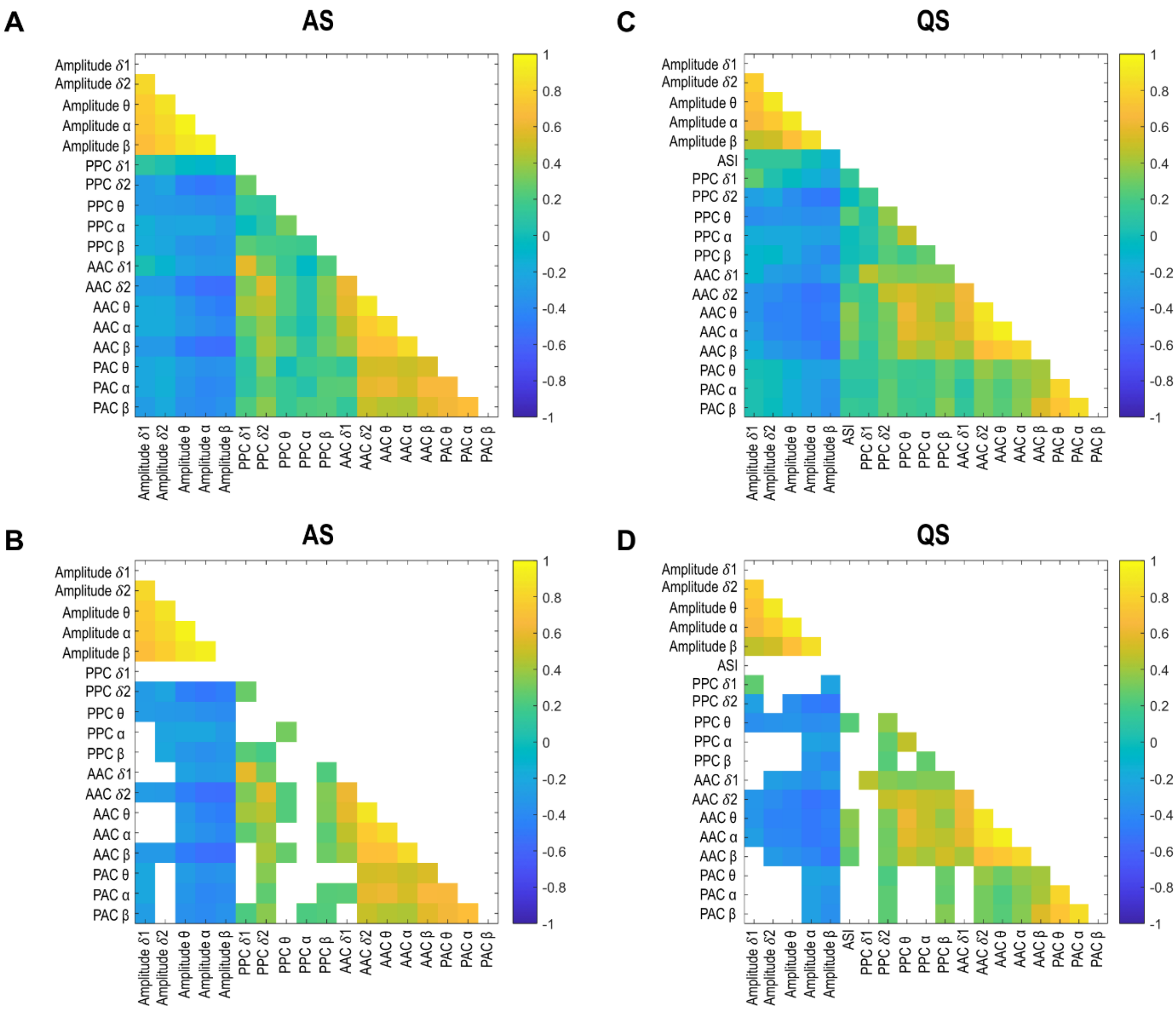

Supplementary Figure 1. Correlations between EEG metrics. Spearman correlation coefficients between all EEG metrics used in this study are shown for A) active sleep and C) quiet sleep, and in B, D) are shown coefficients with $p < 0.05$, respectively.

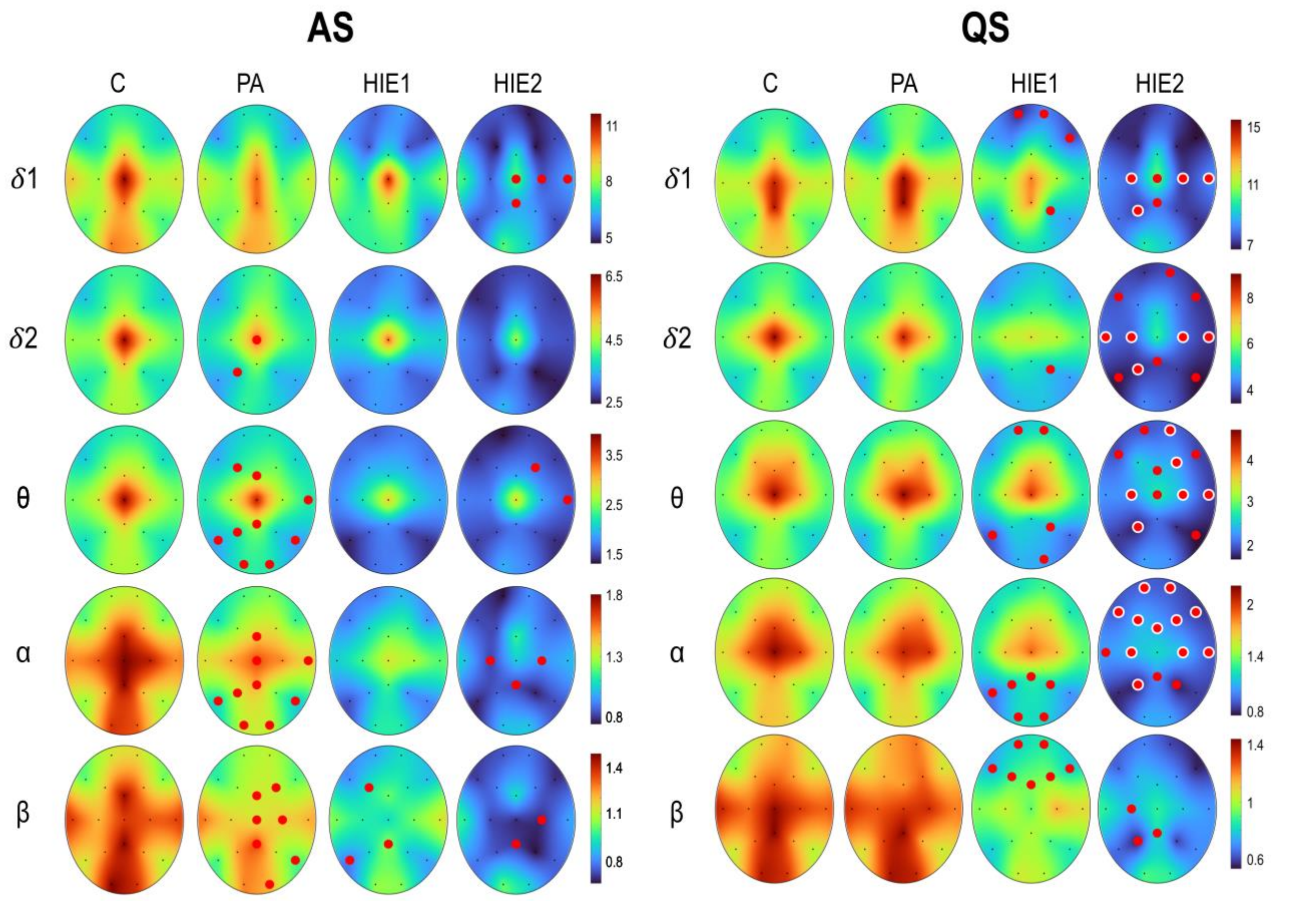

Supplementary Figure 2. Frequency-specific amplitudes in all infant groups. Amplitudes are shown for each group and frequency. Left: active sleep (AS). Right: quiet sleep (QS). Dots indicate difference between adjacent groups. Red dot: group on the left has higher amplitudes in that electrode. Dots are circled with white color if they pass Benjamini-Hochberg correction. δ1: low delta, δ2: high delta, θ: theta, α: alpha, β: beta. C = controls, PA = perinatal asphyxia without HIE, HIE1 = mild hypoxic ischemic encephalopathy, HIE2 = moderate hypoxic ischemic encephalopathy.

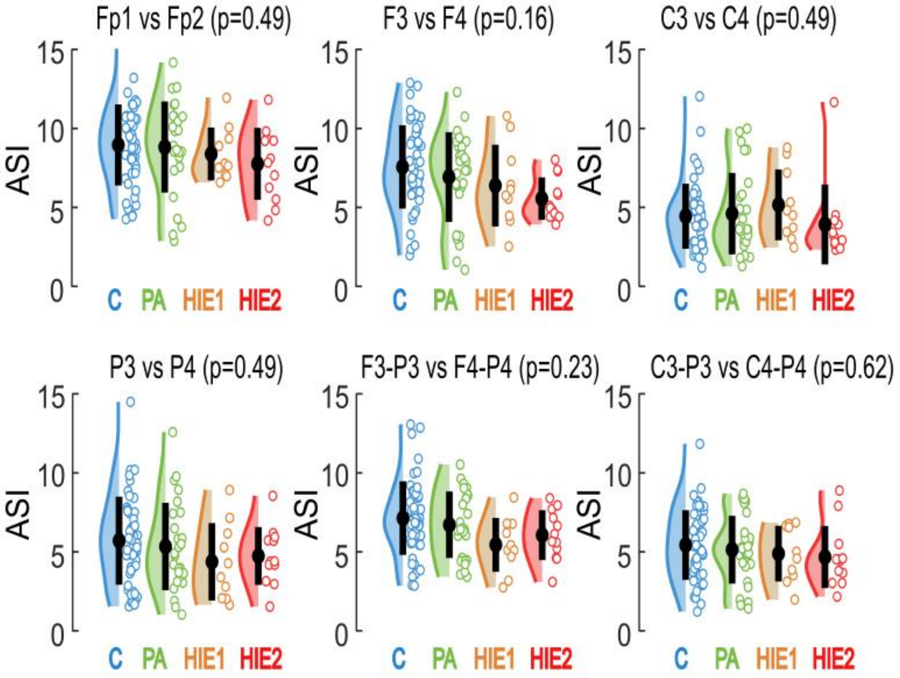

Supplementary Figure 3. ASI comparisons in other electrodes than O1 vs O2. P-values are Benjamini-Hochberg-corrected. C = controls, PA = perinatal asphyxia without HIE, HIE1 = mild hypoxic ischemic encephalopathy, HIE2 = moderate hypoxic ischemic encephalopathy.

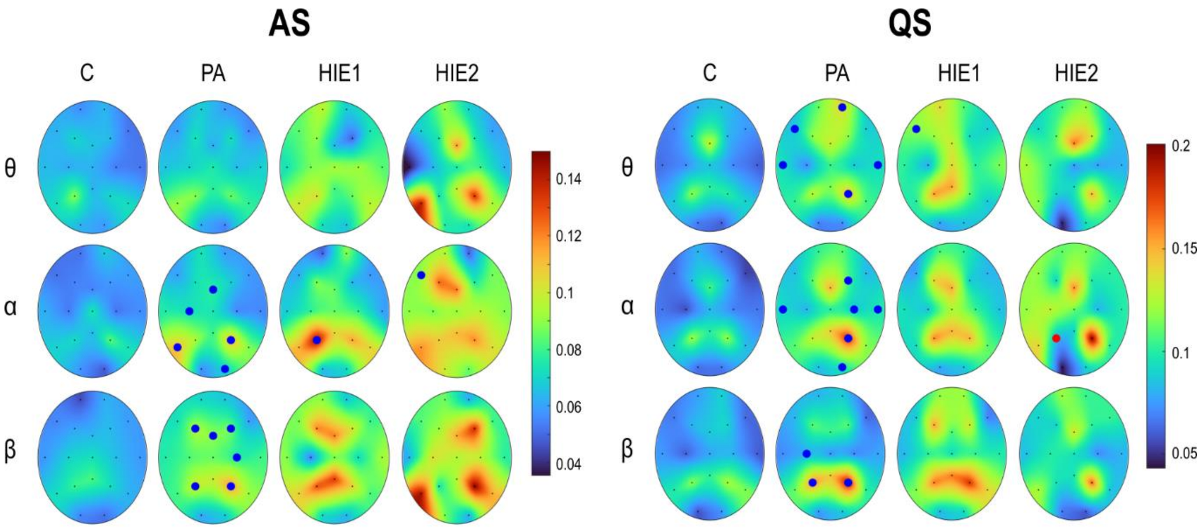

Supplementary Figure 4. Phase-amplitude correlations (PACs) between all infant groups. PACs are shown for each group and frequency. Left: active sleep (AS). Right: quiet sleep (QS). Dots indicate difference between adjacent groups. Red dot: group on the left has higher PACs in that electrode. Blue dot: group on the right has higher PACs in that electrode. None of the dots here pass Benjamini-Hochberg correction. θ: theta (4-8 Hz), α: alpha (8-13 Hz), β: beta (13-22 Hz). C =

controls, PA = perinatal asphyxia without HIE, HIE1 = mild hypoxic ischemic encephalopathy, HIE2 = moderate hypoxic ischemic encephalopathy.

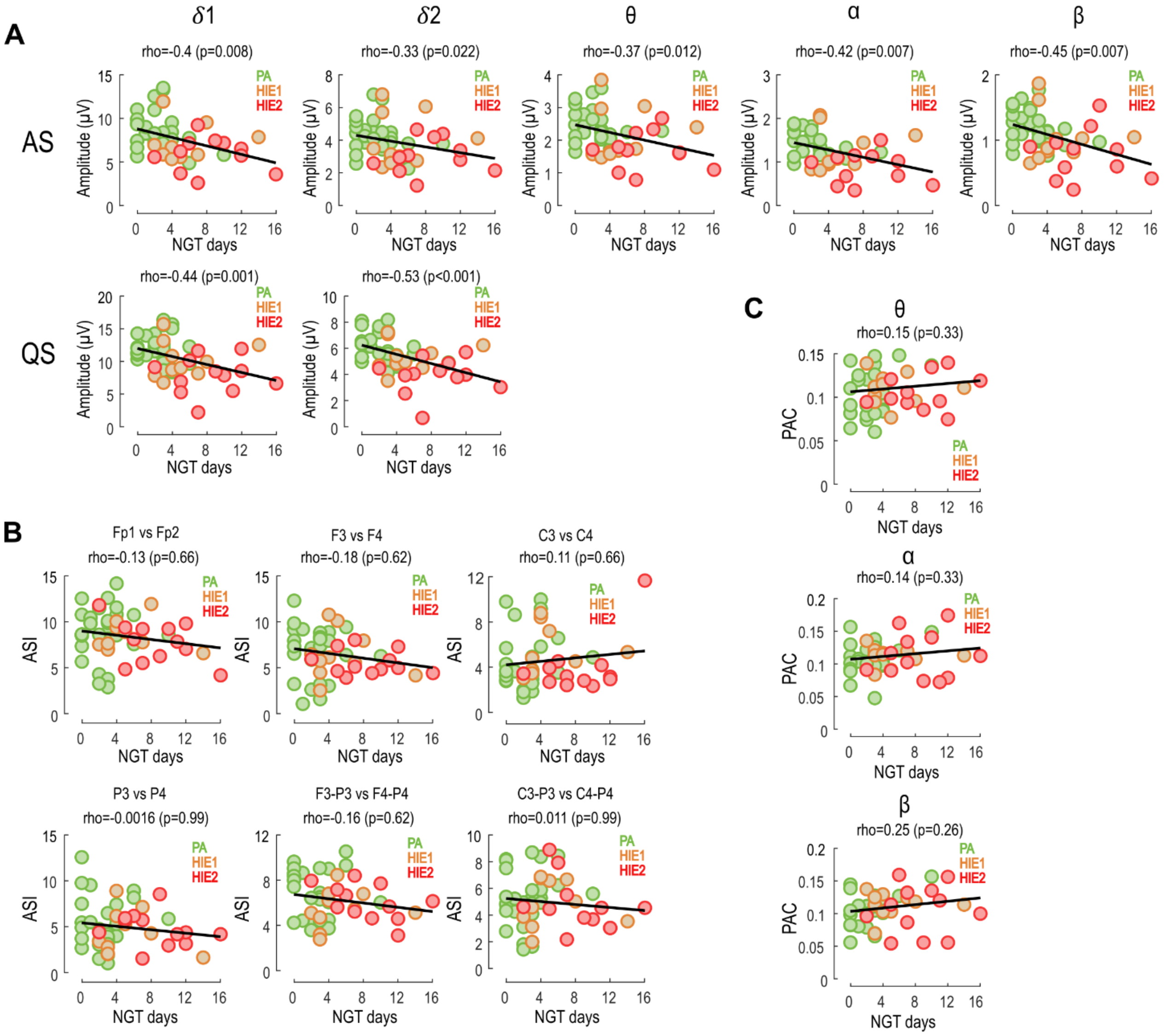

Supplementary Figure 5. EEG metrics and early clinical recovery. Correlations are shown between the nasogastric tube (NGT) removal day and A) amplitudes, B) ASI (other than O1 vs O2 derivation), and C) PACs (in quiet sleep). The dots show individual infants: green = perinatal asphyxia without HIE (PA), yellow = mild HIE (HIE1), red = moderate HIE (HIE2). ρ = Spearman correlation coefficient. δ1: low delta, δ2: high delta, θ: theta, α: alpha, β: beta.
